# Verruciform xanthoma associated with lichen planus

**DOI:** 10.4322/acr.2021.360

**Published:** 2022-02-21

**Authors:** Cristianne Kalinne Santos Medeiros, Glória Maria de França, Weslay Rodrigues da Silva, Joaquim Felipe, Hébel Cavalcanti Galvão, Patrícia Teixeira de Oliveira

**Affiliations:** 1 Universidade Federal do Rio Grande do Norte, Stomatology and Oral Pathology, Natal, RN, Brasil

**Keywords:** Foam cells, Mouth Mucosa, Pathology

## Abstract

Verruciform xanthoma (VX) is a rare benign lesion of unknown etiology, with a rough or papillary aspect, painless, sessile, well-defined, most lesions do not exceed 2 cm in their largest diameter, the degree of keratinization of the surface influences color, varying white to red, affecting mainly the gingiva and alveolar mucosa, and can also be seen in skin and genital**.** Herein, we present a report a clinical case of oral verruciform xanthoma in the buccal mucosa associated with the lichen planus lesion, as well as the morphological and immunohistochemical characteristics of the lesion. The clinical diagnostic hypothesis of oral lichen planus of the white reticular lesions on the buccal mucosa and on the tongue was confirmed by histopathology before a subepithelial connective tissue exhibiting intense inflammatory infiltrate in a predominantly lymphocytic band. In contrast, the hypothesis of the verrucous lesion in the left buccal mucosa was leukoplakia, with histopathological evidence showing exophytic and digitiform proliferations with parakeratin plugs between the papillary projections. Subepithelial connective tissue was characterized by macrophages with foamy cytoplasm (xanthoma cells). An immunohistochemical examination was performed, showing positivity for CD68, a macrophage marker, in addition to testing by Schiff's periodic acid (PAS) with diastasis, which was detected the presence of lipids inside these macrophages. The patient is free of recurrences of verruciform xanthoma and is being monitored due to the presence of lesions of oral lichen planus.

## INTRODUCTION

Verruciform xanthoma (VX) is an uncommon lesion of uncertain etiology, first described in the oral mucosa by Shafer in 1971, but which can also affect the skin and other human mucous membranes.^1^^-^ 4 It is usually diagnosed between the fifth and seventh decade of life, with a slight predilection for males. Clinically, it presents as a sessile, asymptomatic lesion, with a warty, papillary or granular surface, ranging from whitish-yellow to red.^3^^-^ 9 In general, it consists of small lesions located mainly on the gums, tongue, hard palate and buccal mucosa, and a differential diagnosis with other lesions that may affect the oral mucosa is possible, such leukoplakia, hyperkeratosis and white spongy nevus 2 ^,^ 3 ^,^ 5 ^,^ 10 ^,^ 11.

Histopathologically, VX exhibits a stratified squamous epithelium proliferation associated with hyperkeratosis, with uniformly elongated epithelial ridges and the presence of numerous foamy macrophages, of varying sizes and eccentrically positioned nuclei, confined to the papillae of fibrous connective tissue. These cells contain lipids and are known as xanthoma cells.^3^^,^ 5 ^,^ 11 Its pathogenesis is still unclear, although reports of occurrence associated with lichen planus and other immunological and autoimmune diseases are noted, such as pemphigus vulgaris, lupus erythematosus, dystrophic bullous epidermolysis and graft versus host disease, especially when on the skin.^3^^,^ 8 ^,^ 10 ^,^ 12

Rare cases of VX associated with oral lichen planus (OLP) in the oral mucosa have been confirmed through clinical and histopathological examinations. Therefore, present study aims to report a clinical case of VX associated with OLP, addressing clinical, histopathological and immunohistochemical aspects to obtain data to aid in the understanding of its pathogenesis.

## CASE REPORT

**Figure 1:**
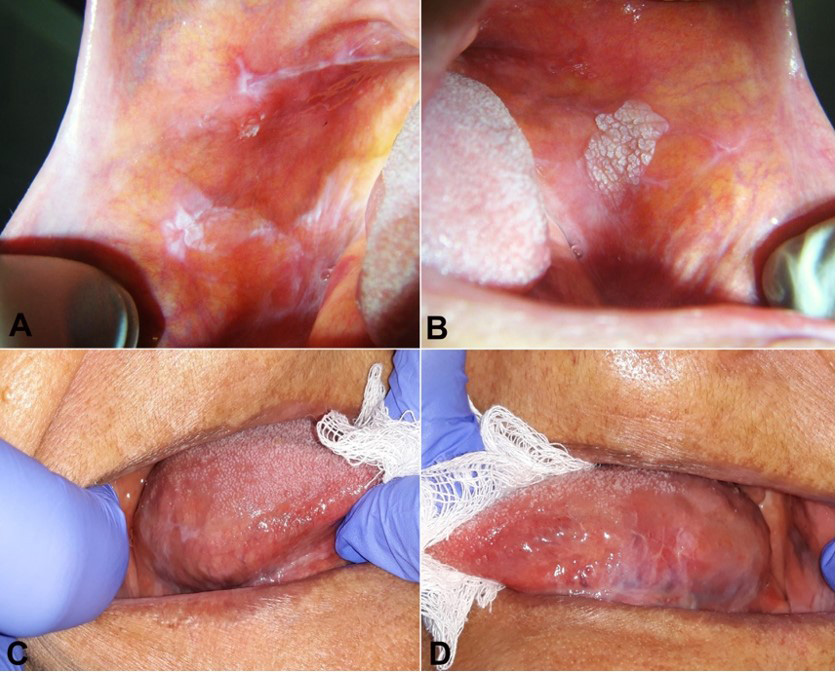
Clinical aspect of lesions clinically diagnosed as OLP and leukoplakia. **A** – white reticular lesions on the right buccal mucosa; **B** – white warty lesion on the left buccal mucosa adjacent to reticular lesions; **C** and **D** – white lesions on the right and left lateral borders of the tongue.

A 74-year-old female patient sought the Stomatology Service in 2019 at Rio Grande do Norte Federal University, in Brazil, complaining of tongue and buccal mucosa lesions, with an unknown evolution time. During the anamnesis, she reported using Risperidone to control anxiety. No history of trauma or smoking habits was noted. Bilateral white reticular lesions were observed on the buccal mucosa, lateral border and inferior tongue face on physical examination, in addition to a white warty plaque with 1.8 cm in size located on the left buccal mucosa, adjacent to the aforementioned reticular lesions (Figure 1).

A clinical diagnosis was OLP in reticular lesions in the bilateral buccal mucosa, lateral border, and inferior tongue face. The warty plaque was clinically diagnosed as leukoplakia. An incisional biopsy was performed on the buccal mucosa on both sides; in the left side, the biopsy involved both the reticular lesion and the warty plaque.

**Figure 2:**
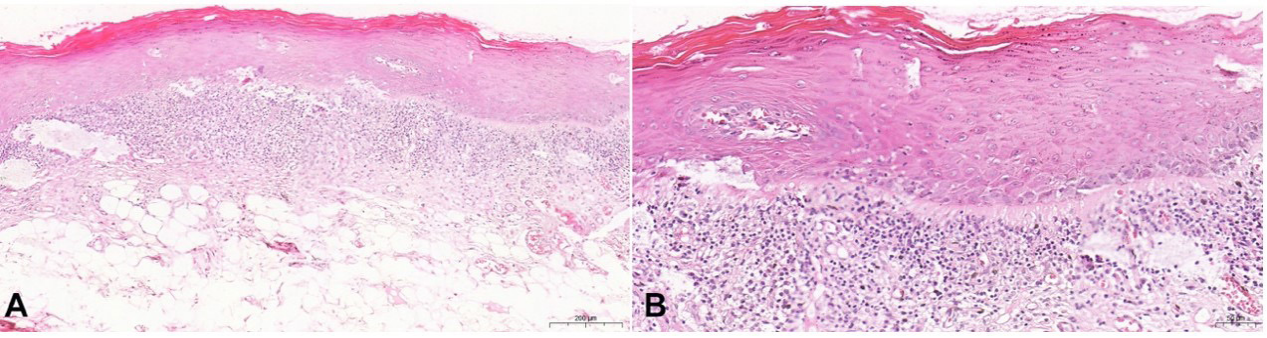
Microscopic characteristics of the OLP, lesion removed from the right buccal mucosa: **A** – lesion covered by stratified hyperortokeratinized squamous epithelium displaying a banded subepithelial inflammatory infiltrate (H&E, scale bar 50 µm; **B** – epithelial changes comprising exocytosis and spongiosis and predominantly lymphocytic connective tissue inflammatory infiltrate )H&E, scale bar 200 µm).

A histopathological examination confirmed the clinical suspicion of OLP for the reticular lesions, in which a fragment of the oral mucosa covered by stratified orthokeratinized squamous epithelium exhibiting acanthosis, spongiosis, exocytosis and edematous degeneration was evidenced. The subepithelial connective tissue presented an intense banded inflammatory infiltrate, predominantly lymphocytic (Figure 2).

**Figure 3:**
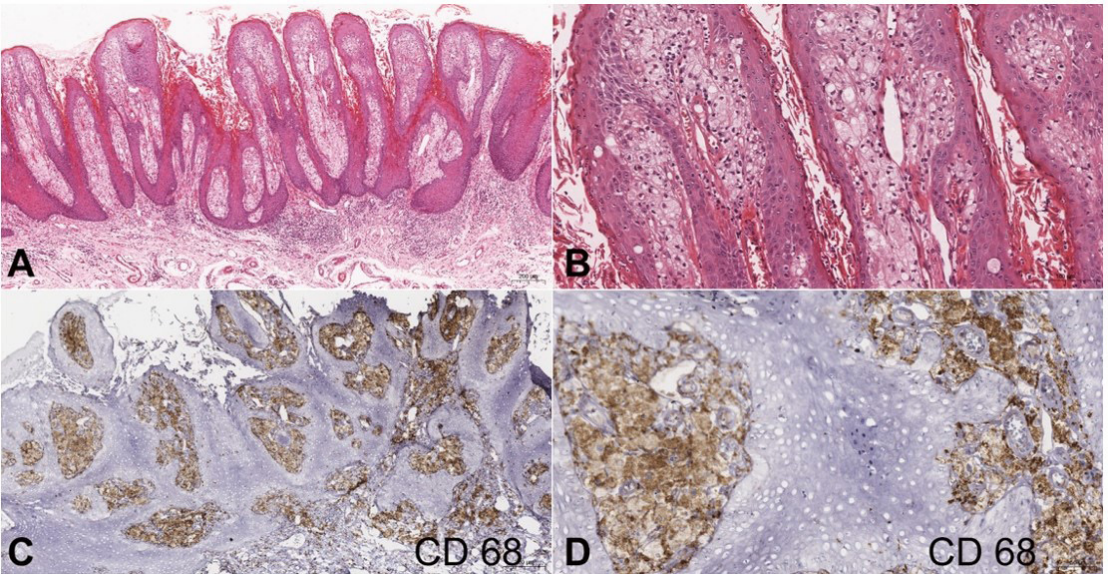
Microscopic characteristics of the VX, lesion removed from the left buccal mucosa: **A** – digitiform proliferation of the stratified parakeratinized squamous epithelium (H&E scale bar 200 µm; **B** – presence of spongy or xanthoma cells ( H&E; scale bar 100 µm); **C** and **D** – positive CD68 immunoexpression in spongy cells (C scale bar 200 µm; D scale bar 50 µm).

**Figure 4:**
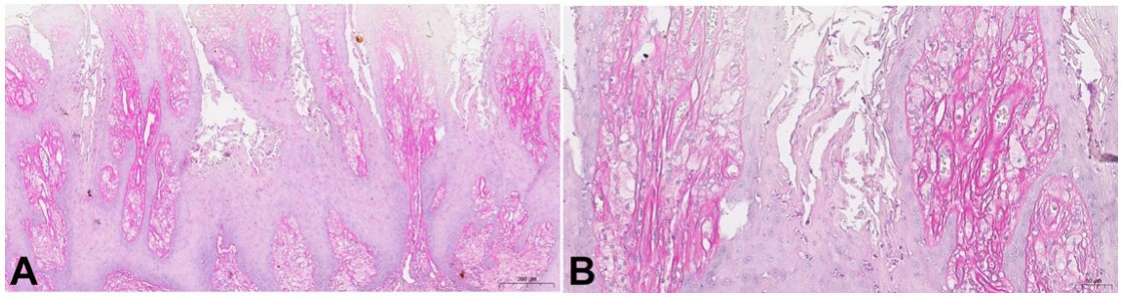
Microscopic characteristics of the VX, lesion removed from the left buccal mucosa: A and B – positivity for PAS with diastasis. (A scale bar 200 µm; B scale bar 50 µm).

The lesion was initially diagnosed as leukoplakia, a hyperparakeratinized stratified squamous epithelium with exophytic and digitiform projections displaying parakeratin plugs. The subepithelial connective tissue presented large spongy cells similar to macrophages. Due to a histopathological suspicion of VX, immunohistochemistry for CD68 and PAS with diastasis was performed, to identify spongy cells and the presence of lipids. Both were positive and a VX diagnosis was confirmed (Figure 3 and 4).

In view of these results, total VX removal was performed, and the patient is currently undergoing clinical follow-up for 18 months.

## DISCUSSION

VX is an asymptomatic, rare, benign soft-tissue growth, and its etiopathogenesis has not yet been clarified. Among the hypotheses suggested for its development is chronic irritation caused by trauma, which would favor epithelial cell degeneration and consequent release of lipid material, which would then be phagocyted by macrophages.^3^^,^ 6 ^,^ 10 ^,^ 13 ^,^ 14 This hypothesis is mainly considered when the lesions in oral mucosa regions more susceptible to trauma, such as the buccal mucosa. In the present case, VX was located in the buccal mucosa, although agravating factors or trauma in the region were not identified, thus discarding this possible etiology. Contrary to the hypothesis of trauma-associated VX pathogenesis, this lesion also occurs in the oral mucosa regions not frequently subjected to trauma.^9^

The presence of periodontal disease pathogens, food allergies and tobacco, alcohol and drug use has also been associated with VX development,^15^ although none of these conditions was observed or reported by the patient in the present case, also discarding this possible etiology.

Another important point related to VX pathogenesis is its association with immunological diseases. Coexistence with lichen sclerosus and lichen has been well described in the literature.^16^ In PubMed, only 16 cases have been reported, associating VX with OLP in the oral mucosa, including the present case.^6^^,^ 8 ^,^ 10 ^,^ 12 ^,^ 15 ^,^ 17 ^-^ 22 It stands out that the studies of Yu et al.,^10^ Ide et al.^15^ and Andrade et al.,^6^ did not present data about the demographic, clinical, or histologic characteristics of the VX and OLP. For this reason, these three articles were not included in Table 1.

**Table 1:** Reports of verruciform xanthoma occurring in patients with diagnosis or clinical evidence of oral lichen planus in the literature

ref	Number of Cases	Age/Sex	Site	Size (cm)	Histological pattern	Association of VX with OLP lesions	Histological confirmation of VX adjacent to OLP
17	1	67/F	Base of the tongue	1,5	NS	Patient with history of OLP	-
18	1	55/F	Buccal mucosa	1	NS	Clinical evidence of OLP	-
11	1	68/F	Lateral border of the tongue	1,3	Verrucous	VX occurring within OLP lesion	Yes
20	3	65/M,	Gingiva, alveolar mucosa, lateral border of the tongue	NS	Flat (3)	VX occurring within OLP lesion (2) and VX at an independent oral site (1)	Yes (2)
		73/F,		-3			
		42/F					
21	1	70/M	Labial mucosa	NS	Flat	Clinical evidence of lichen planus	-
12	1	68/M	Lateral border of the tongue	0,5	Papillary	VX occurring in the site	-
						adjacent to OLP lesion	
8	1	56/F	Lateral border of the tongue	1	Verrucous	VX occurring within OLP lesion	Yes
22	3	63/M,	Buccal mucosa, hard palate,	NS	NS	Patient with history of OLP (3)	-
		61/M,	floor of mouth				
		51/F					
Index case	1	74/F	Buccal mucosa	1,8	Papillary	VX occurring within OLP lesion	Yes

NS: not specified.

It must be considered that of these cases, only 5 present histopathological confirmation of VX adjacent to OLP, suggesting a relationship between these lesions.

This proximity raises the hypothesis that the OLP inflammatory infiltrate may play a role in VX development. It is believed that the accumulation of T lymphocytes present in OLP leads to basal epithelium layer keratinocyte degeneration and, consequently, chemoattractant expression, attracting macrophages to the submucosa region, which would then play an important role in the epithelial alterations observed in these lesions, such as epithelial hyperplasia and hyperkeratosis.^3^^,^ 6 ^,^ 15 ^,^ 23 This hypothesis could justify the association between VX and LP and other immunological or autoimmune conditions.^3^^,^ 6 ^,^ 8 ^-^ 10 ^,^ 12 ^,^ 15 The patient had associated OLP and VX lesions, we believe that the presence of the chronic OLP inflammatory infiltrate may have triggered epithelial changes and participated in the formation of the VX.

The clinical aspect of VX is not pathognomonic; these lesions may have a verrucous, papillary or granular surface.^8^^,^ 9 ^,^ 14 Because of this, many cases may present leukoplakia, hyperkeratosis and white spongy nevus as a clinical diagnosis hypothesis, as noted herein, or even another oral occurrence verrucous lesion. A definitive diagnosis is only obtained by a biopsy and microscopic analyses.^3^ According to Nowparast et al.,^23^ VX can present three histological subtypes, verrucous, papillary and flat. In the present report, the diagnosis hypothesis was leukoplakia and the lesion had a morphologically papillary aspect, with projections of digitiform epithelium and spongy macrophages in the subepithelial region, being the final diagnosis of VX.

In the case in question, immunohistochemical staining with the CD68 antibody was performed in addition to morphological examinations, which displayed immunoreactivity, indicating that the spongy cells present in the submucosa region were macrophages, representing xanthoma cells. Staining by periodic acid-Schiff (PAS) with diastase was also performed and showed positivity for the material among the lipid vacuoles.

The treatment of choice for VX is complete surgical lesion excision, with a good prognosis and low recurrence rates.^3^^,^ 6 ^,^ 10 The patient has been under clinical follow-up for 18 months, with no changes in the VX surgical removal region or the OLP area either.

VX is an uncommon lesion with still unknown etiopathogenesis, with a differential diagnosis from other oral mucosa verrucous lesions. Histopathological analyses of these lesions are paramount for definitive diagnoses.
